# Light-based therapies and radiodermatitis: a case series report

**DOI:** 10.17179/excli2024-7749

**Published:** 2024-10-18

**Authors:** Talita Oliveira de Lima, Karina Alexandra Batista da Silva Freitas, Karen Aline Batista da Silva, Maria Fernanda Setúbal Destro Rodrigues, Thais Barbosa dos Santos, Illora Aswinkumar Darbar Shimozato, Christiane Pavani, Rebeca Boltes Cecatto

**Affiliations:** 1 Biophotonics-Medicine Post-Graduate Program / Universidade Nove de Julho UNINOVE, Rua Vergueiro 235, São Paulo, 01504-001, Brazil; 2 Hospital das Clínicas da Faculdade de Medicina de Botucatu da Universidade Estadual Paulista (HCFMB-UNESP), São Paulo, 18618-687, Brazil; 3 Rehabilitation Service, Instituto de Reabilitação Lucy Montoro, Hospital das Clínicas da Faculdade de Medicina da Universidade de São Paulo, São Paulo, 01246-903, Brazil

**Keywords:** photobiomodulation, radiodermatitis, photodynamic therapy, radiotherapy, case report, head and neck cancer

## Abstract

The main treatments for cancer are radiotherapy and chemotherapy, but they can generate side effects such as fatigue, myelosuppression, and radiodermatitis. The Multinational Association of Supportive Care in Cancer already recommends the use of laser for radiodermatitis in breast cancer patients. However, in relation to head and neck cancer patients, there is a lack of studies clearly demonstrating clinical effects and identifying the best light parameters for the treatment of radiodermatitis. This study reports on three oncological patients with radiodermatitis treated with light-based therapies to show clinical improvements in lesion grades and to discuss the effects of laser and its parameters. A retrospective report of three head and neck cancer patients with radiodermatitis, treated with photobiomodulation and photodynamic therapy at an outpatient health clinical facility. The Visual Analog Scale, Toxicity Criteria of the Radiation Therapy Oncology Group (RTOG) Scale, and the clinical characteristics of lesions were evaluated before and after a photobiomodulation plus photodynamic therapy protocol. Improvements were observed in cases with RTOG grade III with just 4 treatment sessions required for complete healing of the lesions. The patient with RTOG grade IV required antibiotic therapy, temporary suspension of radiotherapy, and more than 4 light sessions to achieve improvements. None of the patients showed worsening of the lesions, necrosis, or infection after treatment with no adverse effects. Head and neck cancer patients with radiodermatitis treated with phototherapy obtained good results in wound healing and pain relief in a short period. These case reports embody the easy-to-apply implementation of a light protocol in a health facility based on previous scientific evidence with positive results and no short-term side effects. In light of the negative impact on quality of life caused by radiodermatitis, health teams should be encouraged to design research study protocols involving light-based therapies.

## Introduction

Cancer is a growing global health problem. According to the International Agency for Research on Cancer (IARC) GLOBOCAN cancer statistics, in 2020, there were more than 19.3 million of new cases (Ferlay et al., 2021[14]). The main treatments for cancer are radiotherapy (RT) and chemotherapy (QT) but both could generate side effects such as fatigue, alopecia, lack of appetite, skin reactions, myelosuppression, among others (Almansour, 2023[2]).

Radiotherapy uses ionizing radiation to destroy cancer cells, stopping their division and, although widely used, RT has side effects, including Radiodermatitis (RD) (Singh et al., 2016[32]). RD lesions are ranging from mild erythema and pruritus to tissue necrosis. It can cause pain, discomfort, infections and affect body image, self-esteem and social interaction, limiting also cancer treatment (Hegedus et al., 2017[21]). The mechanism of RD development is largely related to the inflammatory response associated with oxidative stress. Cellular damage induced by radiation, especially in the mitotic phase, triggers an inflammatory cascade that leads to the modification of cytokines, changes in the cell cycle and even promotes DNA damage. These changes support the inflammatory cascade and consequently lead to disordered tissue repair (Yang et al., 2020[33]).

The Multinational Association of Supportive Care in Cancer (MASCC) is analyzing several treatments for RD (Finkelstein et al., 2022[15]; Behroozian et al., 2023[4]). There is no clear consensus on the most effective prevention measure or treatment for established radiodermatitis but it includes barriers, films, creams, antiseptic agents, analgesics, oral antibiotics and laser therapy (Behroozian et al., 2023[4]). 

Photobiomodulation (PBM) has been studied to treat RD many years ago (Ramos Rocha et al., 2022[27]). PBM accelerates healing through reactions in cellular metabolism, increasing ATP production and promoting anti-inflammatory, analgesic and healing effects (de Freitas and Hamblin 2016[10]). In 2023, Gobbo et al. published a meta-analysis where controlled studies were identified (Gobbo et al., 2023[18]). Breast and head and neck cancer patients receiving PBM experienced less severe RD than the control groups after 40 Gray (Gy) of RT (grade 3 toxicity: Odds Ratio (OR): 0.57, 95 % CI 0.14-2.22, p=0.42) and at the end of RT (grade 0+1 vs. 2+3 toxicity: OR: 0.28, 95 % CI 0.15-0.53, p<0.0001). RT interruptions due to RD severity were more frequent in the control group (OR: 0.81, 95 % CI 0.10-6.58, p=0.85). The authors concluded that preventive PBM may be protective against the development of severe grades of RD and reduce the frequency of RT interruptions. In another study, the DERMISHEAD study (Robijns et al., 2021[29]), 46 patients with head and neck cancer who underwent RT with or without concomitant chemotherapy were selected to receive treatment with PBM or placebo. In this study, 77.8 % of patients in the control group developing grade II RD compared to 28.3 % in the PBM group. Additionally, there was a 49 % of reduction in severe RD in the PBM group. This randomized trial demonstrated the effectiveness of PBM for the prevention and treatment of RD. In this direction, in the latest consensus of Multinational Association of Supportive Care in Cancer (MASCC) (Behroozian et al., 2023[4]), MASCC is already recommending the use of laser as a preventive therapy, since the beginning of RT for breast cancer patients. However, in relation to head and neck cancer patients and RD, there is a lack of studies clearly demonstrating PBM effects and which are the best light parameters. Therefore, even promising publications on Light Based Therapies in the treatment of wounds, there is no agreement on the best recommendation of light to treat RD wound lesions in head and neck cancer patients (Robijns et al., 2022[28]). 

In parallel, the PDT has also been shown to be effective in treating infected wounds of various etiologies (Zhang et al., 2018[36]; Khorsandi et al., 2022[25]; Zhao et al., 2023[37]). PDT uses photosensitizing substances (PS) activated by adequate light, generating reactive oxygen species that inactivate bacteria, fungi and viruses through oxidative stress, having an anti-infectious action (Youf et al., 2021[34]). PDT has also been widely used as an anti-infectious and antineoplastic therapy (Binnal et al., 2022[7]; de Oliveira et al., 2022[11]). Despite this, there are no studies or consensus evaluating the use of PDT in radiodermatitis.

In this sense, this study reports three cases of head and neck cancer patients with RD treated with Light-based therapies to show its clinical improvement on RD grades to discuss the applicability and best parameters of therapeutic light in the treatment of radiodermatitis. 

## Methods and Analyses

### Study design, protocol registration and recruitment 

This is a retrospective report of three head and neck cancer patients with RD, treated with PBM and/or PDT at a Nursing Outpatient Clinic of a University Hospital. It was registered in the Clinical Trials Database (https://clinicaltrials.gov) with the number NCT05557825. This study complies with the Declaration of Helsinki, and it has already been approved by the Research Ethics Committee of Hospital (number 61694722.9.0000.5411). It was conducted according to the CARE Statement Guideline (Gagnier et al., 2014[16]). The data of participants were collected after properly obtaining consent and signing the Free and Informed Consent term. All data published or made available does not contain sensitive data that identifies the patients. The study has not interfered with the clinical follow-up and medical decisions of the health care team or routine of patients. 

### Study population

The sample consists of medical record data from 3 patients who were referred by a medical oncological team to the nursing facility to treatment of RD acquired during RT treatment for cancer in 2022. These patients are also under the care of radiotherapists and oncological team to conventional standardized treatment for RD at the institution. As a routine, the standard RD treatment includes medical follow-up, skin care, compress with chamomile tea (fresh), neutral moisturizer (Calendula 5 % - manipulated) once or twice a day, and topical hydrogel twice a day. 

### Description of the routines followed in the nursing facility for all patients with side effects of oncological treatment 

All patients referred are accepted into nursing facility, except in case of:

- Presence of active untreated tumor at the site to be treated
- Individuals with a history of photosensitivity to photonic or light therapy
- Patients who have undiagnosed lesions in the treatment region
- Patients using topical photosensitizing medications or creams
- Cognitive, psychiatric or neurological changes that prevent the free understanding of the PBM/PDT therapy
- Patients who refuse the treatment.

The nursing team routinely carries out an introductory medical and oncological history. Moreover, the wounds are photographed to show the evolution of the healing (NIKON DX3000 digital camera with speed 1/4000s). The photo angle and distance are calculated by a professional photographer to standardize the images. 

Moreover, standardized serial assessments are then carried out using:

- The Visual Analogic Scale (VAS) (Hawker et al., 2011[20])
- The Toxicity Criteria of the Radiation Therapy Oncology Group Scale (RTOG Scale) translated and adapted by the Portuguese Oncology Nursing Association (Matos et al., 2015[26])
- Clinical characteristics of RD: the presence of bleeding, odor, exudate, crust, phlyctema, hyperemia, peeling, and granulation tissue.

All assessments are routinely done before the start of the therapy and at the end of the treatment. After completion of therapy, adverse effects are assessed and reported. All patients are monitored and treated concomitantly in the oncology, radiotherapy and other specialties or medical interventions that are necessary.

#### Light-Based Therapy Protocol

After local asepsis of the device and skin with liquid polyhexamethylene biguanide (PHMB) 0.1 % for 10 minutes, therapy is carried out every 48 hours until the total wound healing, regardless of the number of sessions required. The therapeutic PBM parameters used are chosen based on the studies of Zecha et al. (2016[35]), Aguiar et al. (2021[1]) and Robijns et al. (2021[29]). It includes the use of the Red Laser (wavelength 660 nm), with a low-intensity laser equipment DMC™ (THERAPY EC / DMC, São Carlos, Brazil) of 100 mW and a cross-sectional area of the device beam 0.0434 cm². The parameters are 1 to 3 J per point depending on the RTOG Scale values, with equidistant points of 1.0 cm, covering the entire length of the open lesion. The laser was kept perpendicular in contact with the skin. For patients who show signs of skin local infection or who present a RTOG Scale value of III or greater, a photodynamic therapy (PDT) protocol is associated with Methylene Blue (MB) 0.01 % as photosensitizer, irradiation with red laser light (660 nm) at 9 joules per point, with equidistant points of 1.0 cm, covering the entire length of the open lesion with a maximum of 4 applications at minimum intervals of 48 hours.

## Results / Case Series Report

### Data extracted from routine medical records of three patients:

#### Case 1

A 75-year-old woman, retired, diagnosed with supraglottis squamous cell carcinoma staged T3N0M0 was evaluated. She performed RT concomitantly with CT, with a RT regimen of 35 fractions of 70 cGy total. 

**Figure 1:**
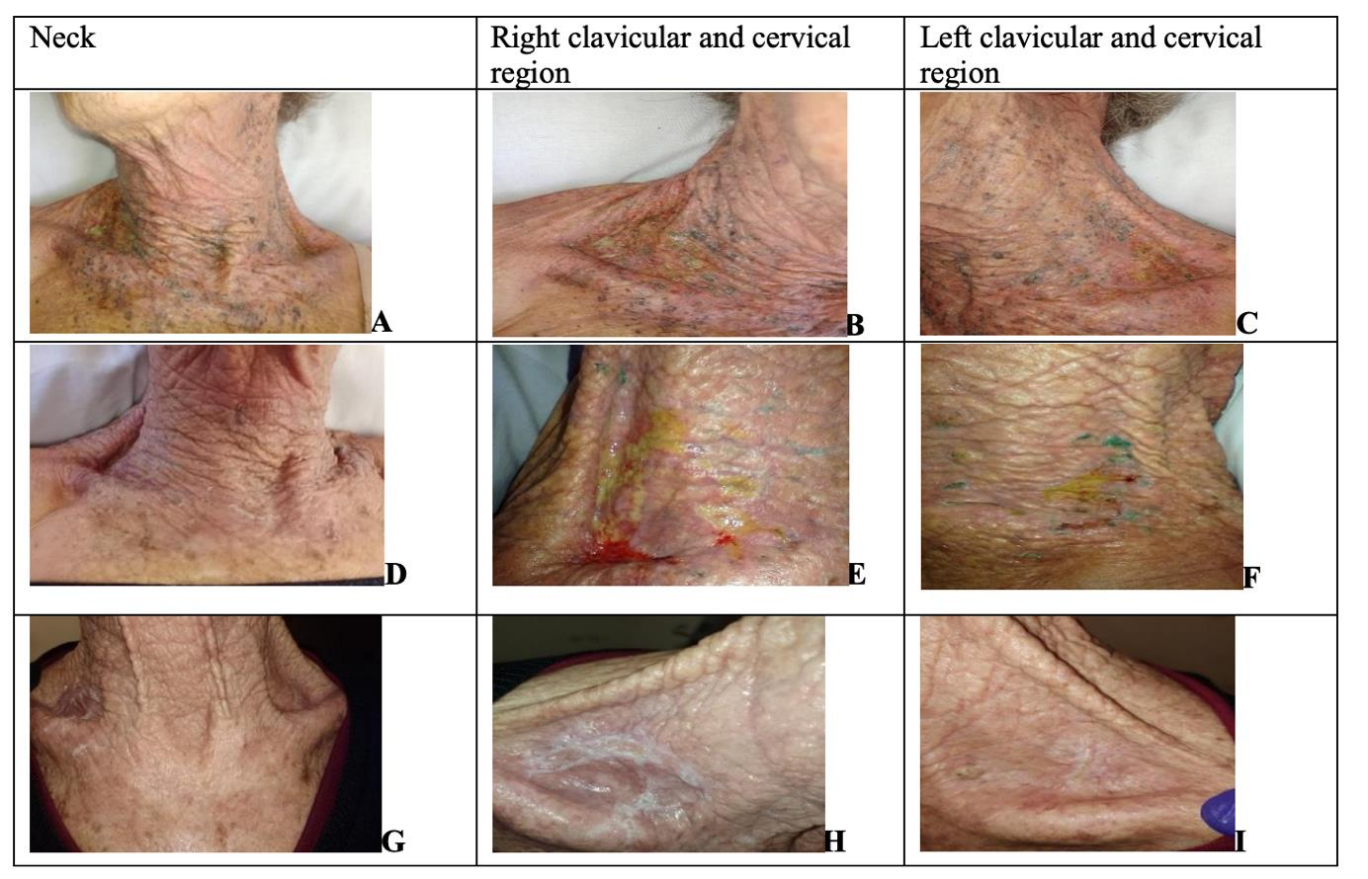
Images of the evolution of wounds after the start of therapy from patient 1. A, B, C are 1^st^ assessment; D, E, F are 5^th^ assessment and G, H, I are 10^th^ assessment.

On the 20^th^ RT session, she was referred to the nursing facility by radiotherapist for evaluation of the lesions that it had started on the 15^th^ RT session. During the evaluation, linear lesions were observed on the right neck and left clavicular region extending to the cervical region, presenting measurements in the left clavicular region of 9.5 cm x 6.3 cm and in the right clavicular region of 9.4 cm x 7.6 cm. The supraclavicular region showed radiodermatitis grade IV on RTOG Scale (G IV), with local hyperemia, presence of exudate in a medium amount of yellowish color, pain (initial of VAS 10), and odor and signs suggestive of local infection (Figure 1(Fig. 1)). The oncological team decided also to add antibiotic therapy and temporarily interrupt RT. A total of 10 treatment light sessions were conducted for this patient during which PDT was administered in 1st, 3rd, 4th, and 6th laser therapy sessions, while PBM was applied in the remaining laser therapy sessions. PBM sessions were performed to achieve complete healing and decreased pain (final VAS of 3). There were no adverse effects of light therapies during the follow-up. 

#### Case 2

**Figure 2:**
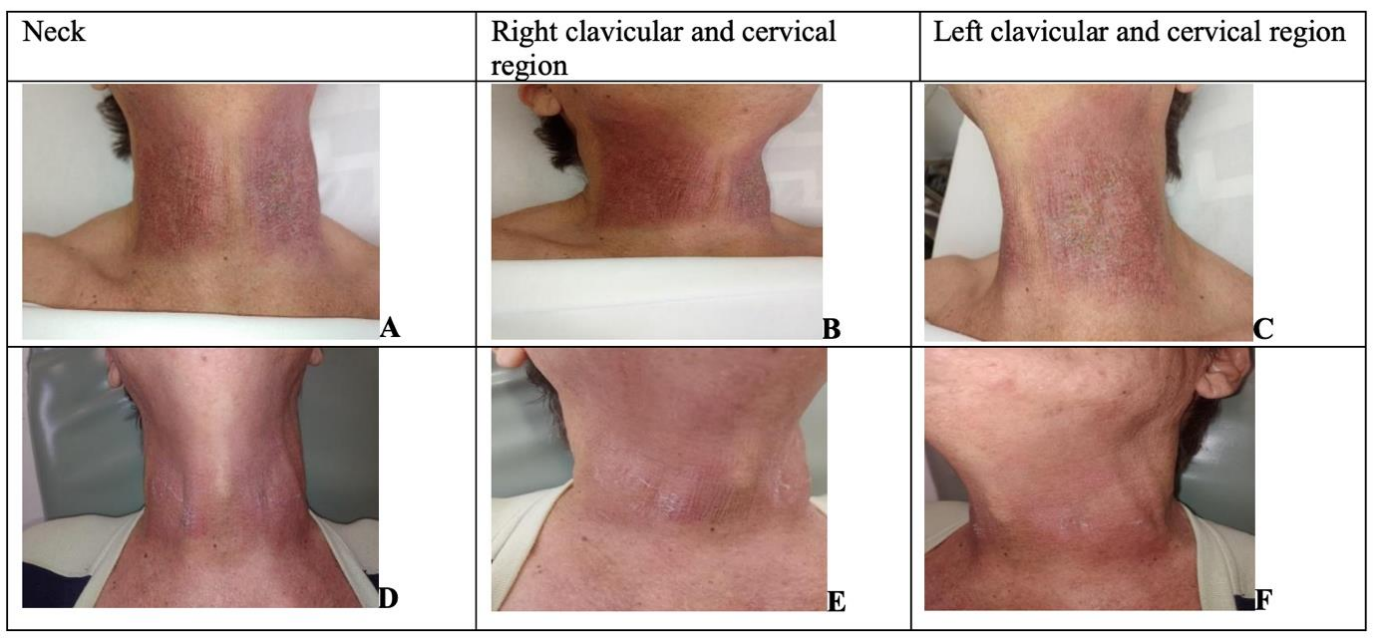
Figure 2: Images of the evolution of wounds after the start of therapy from patient 2. A, B, C are 1^st^ assessment; D, E, F are 5^th^ assessment.

A 65-year-old man, diagnosed with Glottic Squamous Cell Carcinoma extending to the supraglottis with staging T2N0MX was followed. The treatment focused exclusively on RT with a therapeutic regimen totaling 28 fractions, with a total dose of 63 Gy. On the 27th session, linear lesions were observed in cervical and clavicular region, classified as RD grade III (GIII) on RTOG, absence of local secretion, with poorly adherent crust, generalized hyperemia, with moderate desquamation on the left, pain (initial VAS 2) without edema. The lesions presented measurements in the left clavicular region of 10.7 cm x 7.2 cm and in the right clavicular region of 10.5 cm x 8.4 cm (Figure 2(Fig. 2)). Only four light therapy sessions were performed to achieve complete healing and disappearance of pain (final VAS of 0), with discharge. There were no adverse effects observed during the therapy period. 

#### Case 3

A 43-year-old man, carrier of the Human Immunodeficiency Virus (HIV), and diagnosed with oropharyngeal squamous cell carcinoma extending to the supraglottis and staged T3N3M0 was followed. He started RT with a therapeutic regimen totaling 25 fractions of 45 cGy, concomitantly with chemotherapy.

**Figure 3:**
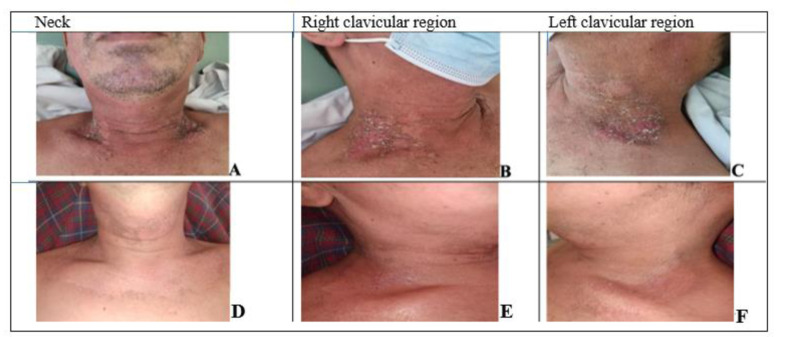
Images of the evolution of wounds after the start of therapy from patient 3. A, B, C are 1^st^ assessment; D, E, F are 3^rd^ assessment.

On the 29^th^ day of RT, the patient attended the laser therapy outpatient clinic for evaluation, and linear lesions were observed in the right cervical region as well as in the supraclavicular region, classified as RDT grade III (GIII) on RTOG Scale and pain (initial VAS of 10). The lesions presented measurements in the left supraclavicular region of 9.0 cm x 5.7 cm and in the right supraclavicular region of 5.1 cm x 4.1 cm (Figure 3(Fig. 3)). In the present study, PDT was conducted in the first two sessions of phototherapy, targeting the supraclavicular regions E and D. PBM sessions were performed to achieve complete healing and decreased pain (final VAS of 5), with discharge. There were no adverse effects of light therapies during follow-up. 

## Discussion

This retrospective study reports three cases of RD after RT treated with PBM and PDT, obtaining good results in both wound healing and pain. Furthermore, no adverse effects were reported in the period evaluated, demonstrating short-term safety.

Cancer is common and affects patients' quality of life and social interaction. Radiation therapy is a standard approach for treating cancer. However, radiodermatitis, a side effect of RT, can lead to inflammation that, if untreated, can result in ulcers or necrosis. This complication can impair treatment adherence, cause pain and impact quality of life during and after treatment (Yang et al., 2020[33]). Several institutions have carried out studies on the prevention and treatment of RD, but there is no consensus on the most effective approach, which is a barrier to its treatment. Newer wound treatment technologies are constantly emerging, and in this matter, new light-based therapies have been studied to improve cancer treatment side effects.

In 2018, Hamblin et al. (2018[19]) published an important review on the safety of PBM in cancer, summarizing numerous results and studies in both animals and humans. At that time, the use of PBM in cancer patients was heavily criticized. But in this review, although there are articles suggesting that PBM therapy may be harmful in animal models of tumors, Hamblin et al. (2018[19]) postulate that there are also numerous studies in humans suggesting that light can directly damage the tumor, enhance other anti-tumor therapies, and may stimulate the host's immune system. The mechanism of action of PBM and its possible direct effect on tumor cells is related to the homeostasis between mitochondrial permeability connections and the triggering of cancer cellular apoptosis (Hamblin et al., 2018[19]; da Silva et al., 2023[9]). Moreover, a possible stimulus carried out by PBM to the immune system could strengthen the T cell immune system, dendritic cells, and type I interferons (Hamblin et al., 2018[19]). 

After that, authors such as Bensadoun et al., (2020[6], 2022[5]), Kauark-Fontes et al. (2022[24]), Genot-Klatersky et al. (2020[17]), de Pauli Paglioni et al. (2019[12]), Silveira et al. (2019[31]), Brandão et al. (2018[8]) and Antunes et al. (2017[3]) reinforce the use of PBM in cancer patients, suggesting safety in its use. Recently, in an elegant study, Robijns et al. (2022[28]) published the first long-term-follow-up study describing clinical data of 120 breast cancer patients and RD treated with prophylactic PBM or placebo with a median follow‐up time of 66 months. There was no significant difference in disease-free survival (73.7 % vs. 98.3 %, resp., p= 0.54), cancer-free survival (68.4 % vs.77.8 %, resp., p= 0.79), and overall survival (87.9 % vs. 98.3 %, resp., p= 0.30) between the placebo and PBM group. 

*In vivo* tumor heterogeneity and microenvironment may influence results and justify the contradictions observed in some *in vitro* studies. Clinical trials allow evaluating the effect of PBM on the tumor cells and microenvironment in humans (e.g., immunological function, the surface microenvironment, and epithelial and tissue co-interactions) suggest safety. While additional clinical studies and greater follow-up time to evaluate oncological outcomes as survival, metastasis or recurrence are desirable, current evidence suggests short-term safety of PBM. It seems unlikely that PBM has carcinogenic effects or can shield cancer cells from cancer treatment. 

These concepts, in addition to the characteristics of PBM already known increasing analgesia, tissue repair, and reducing inflammation, have supported numerous studies and protocols aiming the use of PBM as an adjuvant treatment for complications of oncological treatment. It includes lymphedema, pain, chemotherapy-induced peripheral neuropathy, and oral mucositis among others (de Pauli Paglioni et al., 2019[12]; Bensadoun et al., 2020[6], 2022[5]; Robijns et al., 2021[29]). In this sense, research encouraging the use of PBM in the prevention and treatment of radiodermatitis is growing up, considering its effectiveness in treating and preventing other side effects of oncological treatment, such as the oral mucositis and lymphedema (Elad et al., 2020[13]).

In parallel, although scientific studies in the medical literature recognize that randomized controlled clinical trials are the gold standard for evaluating treatments, case series still form a large part of the scientific publication. In addition, many issues related to healthcare practice and clinical implementation of these protocols, such as the number of sessions required, therapeutic parameters, impact on quality of life, patient satisfaction, minimum clinical effect, possible discomforts of therapy, or treatment costs, are still unknown and have a profound impact on the implementation of knowledge arising from randomized clinical studies. A research-to-practice gap is still present in traditional healthcare researchers. Therefore, case series are key points to health professional team knowledge because they often replicate their experience and are able to produce clinical insights regarding issues related to daily care practice and its difficulties. They often provide information on new treatments and important preliminary data necessary for randomized clinical trials. Furthermore, for uncommon therapies, center's experience may be real models of implemented science. 

In this case series report, improvements were observed in 2 of 3 cases with grading (RTOG III) with only 4 treatment sessions to complete healing of the lesions. The patient classified with RTOG IV grade, required suspension of radiotherapy, antibiotic therapy and plus 10 light therapy sessions. Despite this difference, none of the patients treated in this protocol showed worsening of the lesions or emergence of necrosis or local infection after the introduction of light-based therapy, which is normally very common in patients with RD that appears concomitantly with RT. These aspects corroborate the efficacy of the light therapies used in these patients and suggest that PBM and PDT are promising techniques for wound lesions, and can prevent RD worsening, the process of local skin infection and accelerate the healing of active lesions. For patients with RTOG III a small number of sessions were sufficient to achieve good results. In a pioneering study similar to our report, Hottz et al. (2022[22]) published a case report of a 62-year-old man with grade 3 RTOG perianal radiodermatitis, which was related to radiotherapy for an invasive anal squamous cell carcinoma. The radiodermatitis lesions were treated with a red light PBM protocol (2 J, twice a week, for 4 weeks, until D7 after RT completion), which resulted in the relief of pain and burning symptoms, as evaluated by a VAS score reduction from 9 to 3, and a decrease in the radiodermatitis grade from 3 to 2 RTOG. The relief of radiodermatitis symptoms occurred after the first application. By the seventh PBM application, the patient reported a VAS score of 3, and by the eighth application, the patient was asymptomatic. This outcome corroborates our findings and reinforces the conclusion that PBM effects can be achieved with a small number of sessions, even in patients with signs of already established RD and RTOG 3. Furthermore, it is worth highlighting that the previous studies analyzed in the literature demonstrating the effectiveness of PBM applied since the beginning of RT, before the formation of lesions, showing the action of light in a protective way on the tissue even with few number of light sessions (Ramos Rocha et al., 2022[27]; Behroozian et al., 2023[4]; Gobbo et al., 2023[18]). 

This study has limitations. First of all, the three patients continued to use standardized therapy in the hospital. Another important point of the protocol was the associated use of the PHMB to clean the lesions before light therapy. Its action is based on the interaction with phospholipids in the bacterial membrane, leading to rupture, and the inhibition of bacterial metabolism (Hübner and Kramer, 2010[23]) therefore, it removes mechanical barriers that interfere with tissue interaction with light and/or photosensitizer which may have amplified the results of phototherapy. Therefore, it is not possible to state that the benefits found after light treatment come exclusively from PDT or PBM.

However, in general, RD injuries that appear concomitantly with RT treatment are time-consuming and require several weeks for healing. They tend to worsen and not stabilize or regress during the period while RT is active (Singh et al., 2016[32]; Hegedus et al., 2017[21]; Yang et al., 2020[33]). In this sense these results suggest an effective action of light therapy on healing of RD in head and neck cancer patients even with active RD lesions.

### Implications for clinical practice

Although randomized clinical studies or literature reviews have already evaluated the effects of light therapies for treating the adverse effects of radiotherapy, few studies discussing issues related to the operationalization of these protocols such as the number of sessions required or impact on satisfaction of patients. MASCC is already recommending the use of PBM as a preventive therapy to RD, since the beginning of RT for breast cancer patients (Behroozian et al., 2023[4]). But in relation to head and neck cancer patients there is a lack of studies clearly demonstrating the results and which are the best light parameters. This study moves in these directions and evaluate the results of a model of nursing practice on oncological head and neck cancer patients with RD, bringing future contributions to the development of studies on radiodermatitis supportive treatment. Taking into account the great negative impact on quality of life caused by RD, health teams in the support of cancer patients should be encouraged to discuss studies that incorporate light-based therapies. Our case reports embody the easy-to-apply implementation of a light protocol based on previous scientific evidence and demonstrate positive results without short-term side effects in this matter.

## Conclusion

This retrospective study suggests that the Light-Based Therapy Protocol described here associated to standard treatment is a promising technique in the supportive therapy of RD lesions, and, could accelerate the healing of active lesions in a fast time therapy, even in head and neck cancer patients during radiotherapy follow-up. For patients with RTOG III classification, just 4 treatment sessions were sufficient to achieve good results. For patients with RTOG IV grade, future studies evaluating cost/benefit and minimum treatment time are necessary. The results of this study corroborate previous RCT studies demonstrating that PBM, as well as PDT, are safe techniques in a short-term follow-up and can be studied as a supportive effective therapy in treating side effects of radiotherapy on oncological patients.

## Declaration

### Reporting method

This study adhered to CARE Statement Guideline. 

### Ethical considerations

This study complies with the Declaration of Helsinki, and it has already been approved by the ethics committee by the Hospital das Clinicas da Universidade UNESP BOTUCATU Research Ethics Committee (number 61694722.9.0000.5411). It was conducted according to the CARE Statement Guideline.

### Patient consent statement

All participants included in this study permit and consent the publication of data. The data of participants were collected after properly obtaining consent and signing the Free and Informed Consent term. All data published or made available does not contain sensitive data that identify the patients. The study has not interfered with the clinical follow-up and medical decisions of the health care team or routine of patients. 

### Consent for reproduced material from other sources

Not applicable. This study did not use, reproduce or translate instruments, software or other tools from other sources that require formal permission.

### Clinical Trials Database

This study protocol is previously registered on Clinical Trials Database under the number NCT05557825 (https://clinicaltrials.gov/search?intr=NCT05557825%20. 

### Author contributions

**TOL:** Conceptualization, writing - original draft, methodology, data acquisition, formal analysis, writing - review & editing. **KABSF: **Investigation, formal analysis, data acquisition, writing - review & editing; **KABS:** Resources, writing - review & editing, validation. **MFDSR**: Methodology, resources, writing - review & editing, validation. **TBS:** Resources, writing - review & editing, validation.** IADS:** Resources, writing - review & editing, validation.** CP**: Methodology, resources, writing - review & editing**; **validation. **RBC: **Conceptualization, methodology, data curation, supervision, resources, validation, writing - review & editing, project administration.

All authors reviewed and approved the final version of the manuscript.

### Data availability statement

All data relating to this study are presented in the manuscript.

### Disclosure/Competing of interest 

The authors have no relevant financial or non-financial conflicts of interest to disclose. The author(s) declared no potential conflicts of interest with respect to research, authorship, and/or publication of this article.

### Funding

Programa de Excelência Acadêmica (PROEX) da Coordenação de Aperfeiçoamento de Pessoal de Nível Superior (CAPES)/ Brazil, granted through a Scholarship from the Post-Graduate Program at Universidade Nove de Julho, without any influence on the impartial conduct of the research to Talita Oliveira Lima (88887.864230/2023-00) and to Illora A. D. Shimozato (88887.976667/2024-00).

### Sponsor

Universidade Nove de Julho / UNINOVE provided consumables, internet access, databases and bibliographic support, physical space and digital resources to carry out this study.
